# A rare cause of back pain and radiculopathy – spinal tophi: a case report

**DOI:** 10.1186/s13256-018-1940-4

**Published:** 2019-01-08

**Authors:** S. A. Wan, C. L. Teh, A. T. Jobli, Y. K. Cheong, W. V. Chin, B. B. Tan

**Affiliations:** 1 Rheumatology Unit, Sarawak General Hospital, Jalan Hospital, 93586 Kuching, Sarawak Malaysia; 2 Radiology Department, Universiti Malaysia Sarawak, 94300 Kota Samarahan, Sarawak Malaysia; 3 Orthopaedics Department, Universiti Malaysia Sarawak, 94300 Kota Samarahan, Sarawak Malaysia

**Keywords:** Spinal tophi, Axial gout, Gout

## Abstract

**Background:**

Gout is a monosodium urate deposition disease which is prevalent worldwide. The usual manifestations are crystal arthropathy and tophi deposition in the soft tissues. Spinal tophi may also occur and are rarely reported, resulting in various clinical manifestations such as back pain, spinal cord compression, radiculopathy, and even mimicking epidural abscess and spondylodiscitis.

**Case presentation:**

We report a case of a 42-year-old Chinese man with underlying gout who presented with back pain and radiculopathy. The diagnosis of spinal tophi was unsuspected and he was initially treated for epidural abscess and spondylodiscitis. He underwent a laminectomy and posterolateral fusion during which tophus material was discovered. He recovered and medications for gout were started.

**Conclusion:**

Spinal tophi are rare. The diagnosis is difficult and spinal tophi may be mistaken for epidural abscess, spondylodiscitis, or neoplasm.

## Background

Gout is a monosodium urate deposition disease [1]. Deposition of monosodium urate crystals in the joints result in gouty arthritis and deposition of monosodium urate crystals in the soft tissue result in tophi formation. Although gout is prevalent in society, spinal gout is rarely reported. Spinal tophi are not usually suspected when a patient presents with back pain with neurological symptoms. Spinal cord compression, epidural abscess, spondylodiscitis, and malignancy are more common diagnoses. We present a case of a patient with back pain with radiculopathy due to spinal tophi.

## Case presentation

We report the case of a 42-year-old Chinese man with a history of chronic tophaceous gout who presented with back pain 2 years ago. The pain was sudden, located at his lower back, radiated to his left lower limb, persisted for a few days, and was subsequently relieved with non-steroidal anti-inflammatory drugs (NSAIDs). There were no neurological abnormalities at that time and further investigations were not performed. He continued to experience episodes of the same back pain over the next 18 months. Two months prior to hospitalization, he had another episode of severe back pain which radiated down to his left lower limb with weakness of his left lower limb. There was no history of trauma, prolonged fever, cough, hemoptysis, loss of appetite, loss of weight, or incontinence.

His past medical history included gout which was diagnosed 4 years ago. He had monthly recurrent gouty arthritis, which affected his first metatarsophalangeal joints, ankles, knees, and shoulders. He noted multiple swellings over his limbs for the past 3 years. During this period, he self-medicated with NSAIDs which terminated the gouty arthritis episodes. He did not seek any medical treatment for urate-lowering therapy.

A physical examination showed normal cardiovascular, respiratory, and abdominal systems. There were multiple tophi seen over the dorsum of bilateral hands, bilateral elbows, bilateral ankles, and toes. A neurological examination showed normal tone in his bilateral lower limbs. Power was reduced for left thigh flexion and extension (3/5) and knee flexion (4/5). His left knee jerk reflex and left ankle jerk reflex were reduced. Sensation was reduced at left L4 and L5 dermatomes. There was no sensory level. His anal tone was normal. Neurology of his upper limbs was normal.

**Fig. 1:**
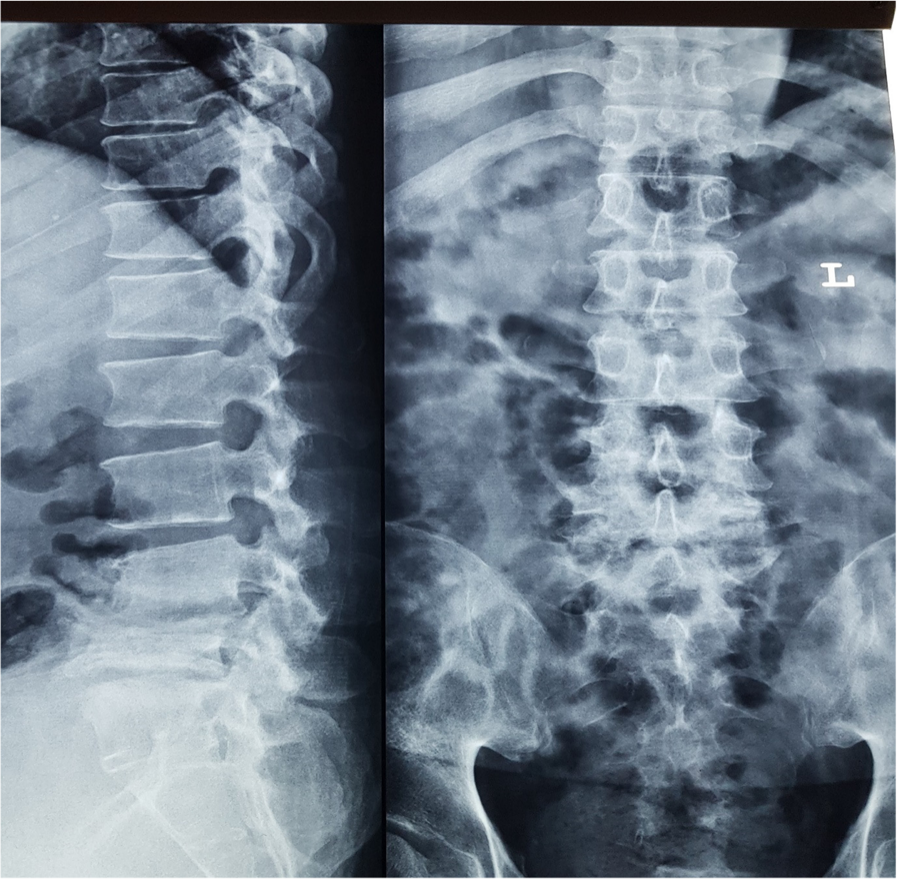
Lumbosacral radiograph showing irregularities of the L4, L5, and S1 endplate with reduction in L4/L5 and L5/S1 intervertebral discs space and L5 vertebral body

**Fig. 2:**
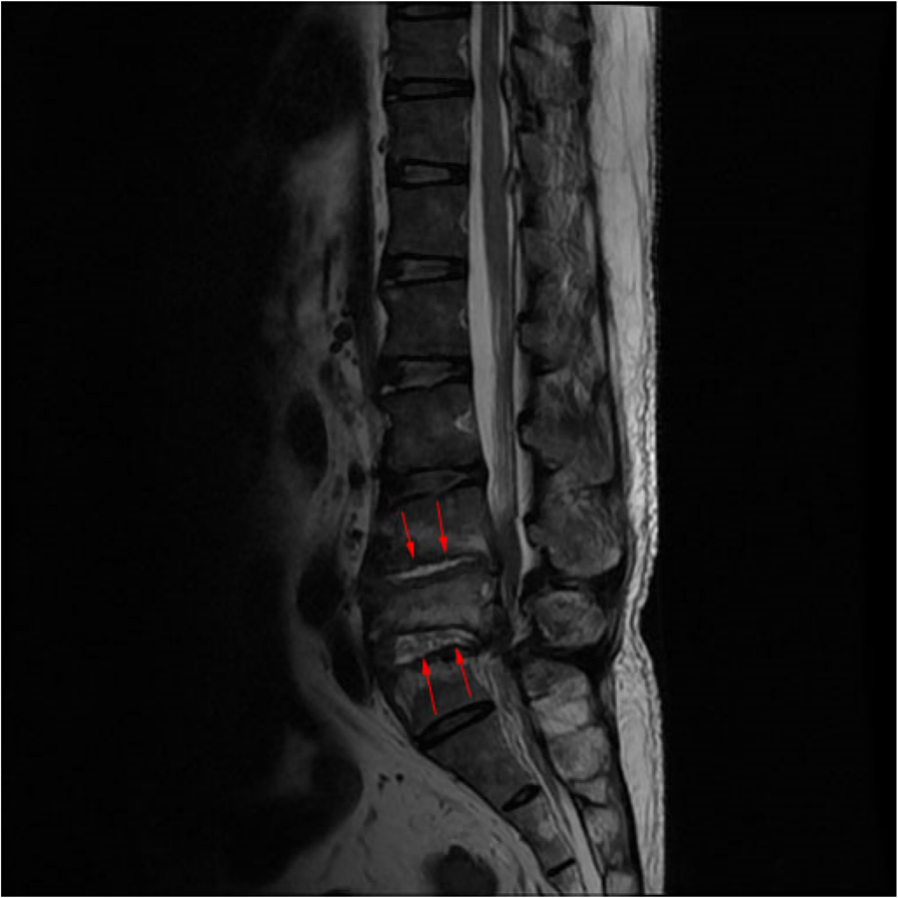
Hyperintensity within the intervertebral discs spaces of L4/L5 and L5/S1 on T2-weighted imaging in keeping with fluid within. Fluid within the L4/L5 and L5/S1 intervertebral discs spaces (*arrows*) on T2-weighted imaging

**Fig. 3:**
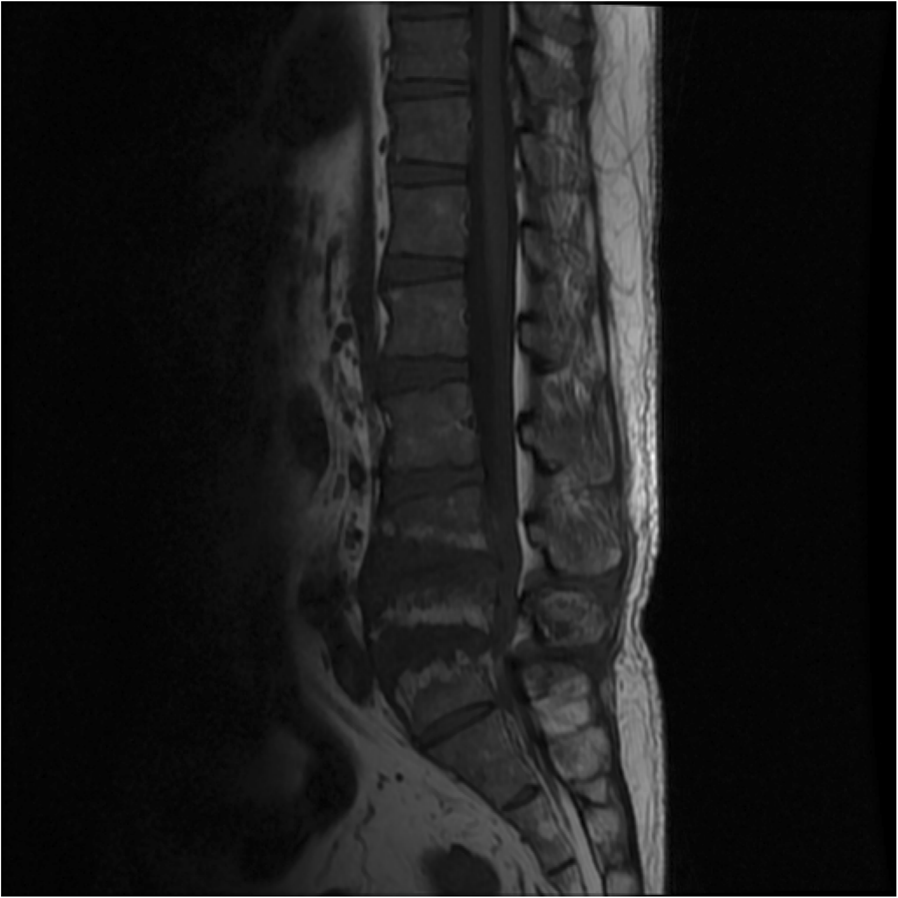
Irregular endplate erosion manifested as hypointensity on T1-weighted imaging

**Fig. 4:**
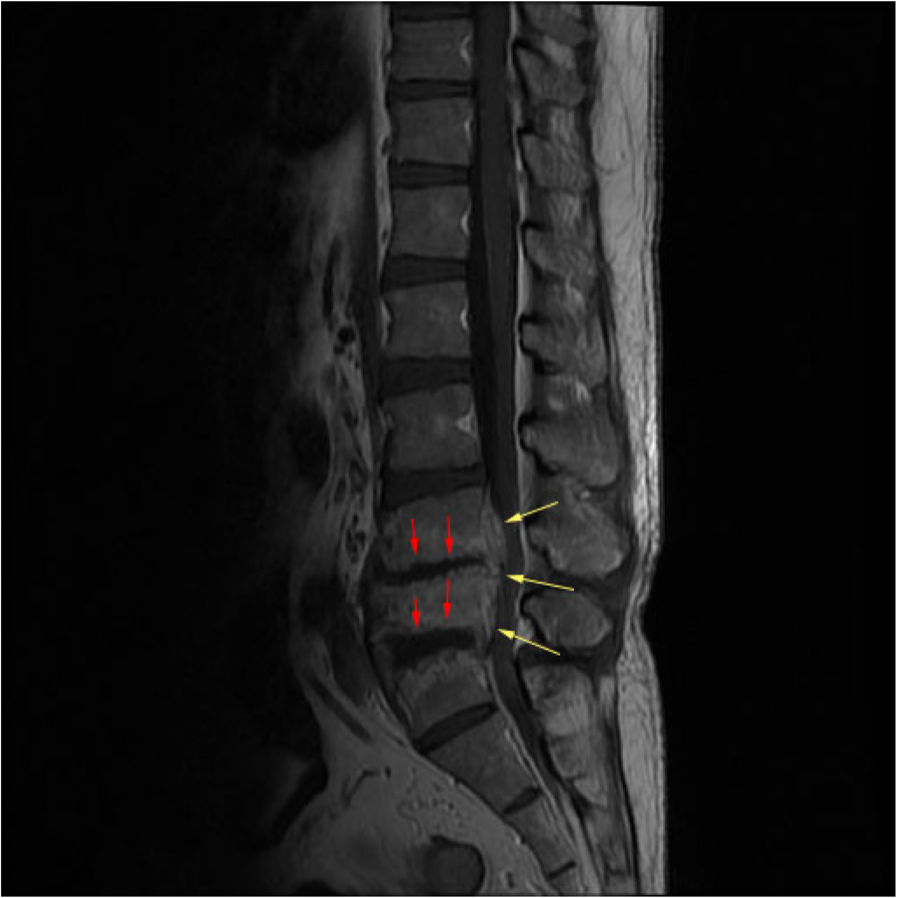
T1-weighted imaging post contrast showing enhancement of the irregular endplate (*red arrows*) with enhancing epidural mass-like lesion (*yellow arrows*)

Full blood count: total white cell, 18 × 10^3^/μL (3.99–10); hemoglobin, 11 g/dL (12.1–18.1); platelets, 526 × 10^3^/μL (142–424). Creatinine was 165 μmol/L (60–120). Creatinine clearance was 58 ml/minute. Sodium was 130 mmol/L (135–145), potassium was 3.2 mmol/L (3.3–5.1), and urea was 7.6 mmol/L (1.7–8.3). Uric acid was 524 μmol/L (202–420). C-reactive protein (CRP) positive was 96 mg/L. An echocardiogram showed no vegetations. A chest radiograph was normal. Lumbosacral radiographs showed irregularities of the L4, L5, and S1 endplate with reduction in L4/L5 and L5/S1 intervertebral discs space and L5 vertebral body (Fig. 1). MRI of his spine showed hyperintensity within the intervertebral discs spaces of L4/L5 and L5/S1 on T2-weighted imaging (T2WI) in keeping with fluid within (Fig. 2). There was also irregular endplate erosion manifested as hypointensity on T1-weighted imaging (T1WI; Fig. 3) which demonstrated heterogenous enhancement of the involved vertebral endplate and epidural components post contrast (Fig. 4). The initial diagnosis was epidural abscess with spondylodiscitis. *Staphylococcus aureus* or *Mycobacterium tuberculosis* infection was suspected. He was started on intravenously administered cloxacillin. Investigations for tuberculosis were negative. Blood cultures were negative. A percutaneous biopsy was not performed as the clinical suspicion for epidural abscess and spondylodiscitis was high and the differential diagnoses of tumor and spinal tophi were not suspected. He underwent surgery to drain the abscess and laminectomy and posterolateral fusion.

Operative findings showed chalky white non-adherent material over the facet joints resembling gouty tophi. A small mass lesion with bony erosion was noted over the left L4/L5 facet joint extending and causing a small bony defect on the left side of the L4 lamina. There was no pus or slough seen at the operative site. Pedicle screws were inserted at the desired lumbar and sacral levels, mainly from L3 to S1. Laminectomy was performed at the L4 and L5 levels. Pre-contoured rods were inserted on both sides followed by posterolateral fusion. He was treated for spinal tophi with colchicine.

The vertebral disc was sent for histopathological examination but yielded necrotic tissue only. Tissue cultures were negative, acid-fast bacilli smears were negative, and tuberculosis culture was negative.

During the admission, he had a flare of gouty arthritis of his right wrist and metacarpophalangeal joints. He was started on colchicine and a course of steroids and the gouty arthritis subsequently resolved. During follow up, his back pain improved and he was started on allopurinol for urate-lowering therapy.

## Discussion

Our case demonstrated the difficulty of diagnosis of spinal gouty tophi due to the rarity of the condition. The initial diagnosis was epidural abscess with spondylodiscitis. In this part of the world, tuberculosis of the spine must be considered as well. *Staphylococcus aureus* is the predominant organism causing spondylodiscitis, accounting for half of non-tuberculous cases [2]. Another possible differential diagnosis is neoplasm. The history, physical examination, and spine imaging were pointing toward an infective process, especially with the vertebral destruction seen on the spine radiograph and the MR images. The diagnosis of spinal tophi was not suspected despite our patient having multiple peripheral tophi and having acute polyarticular gout at the time of presentation. Similar case reports have been published in which initial suspected epidural abscess and spondylodiscitis were later found to be spinal tophi after surgery was performed [3–8].

The various clinical presentations of spinal gout were described by Elgafy *et al.* in a review of 68 cases of spinal gout [9]. The most common clinical presentation was back pain, followed by spinal cord compression, spinal nerve root compression, fever, cranial nerve palsy, and atlantoaxial subluxation. The most common location of spinal gout involvement was the lumbar region (38 patients), followed by the thoracic region in 15 patients, and the cervical region in 15 patients. Clinicians usually suspected other conditions such as infections (epidural abscess, spondylodiscitis) or neoplasm before the diagnosis of spinal tophi was established either by fine-needle aspiration or biopsy or during surgery [10–16].

Although spinal gout was thought of as rare, it may be underdiagnosed. Only those with neurological symptoms and back pain will present and be investigated with or without surgical intervention. Other patients who are asymptomatic may not be diagnosed. Konatalapalli *et al.* [17] reviewed 92 patients with gout and 64 had undergone computed tomography (CT) of the spine for various reasons. Out of the 64 patients, nine had features of spinal gout. Spinal gout was diagnosed clinically in one patient. The same group later performed a cross-sectional study to determine the prevalence of spinal gout in patients with gouty arthritis and found that among the 48 patients, 35% had spinal erosions and/or tophi [18], and 15% had spinal tophi. All patients with spinal tophi had abnormal hand or feet radiographs. Konatalapalli *et al*. [18] found that extremity radiographs characteristic of gouty arthropathy correlated strongly with CT evidence of spinal gout. Duration of gout, presence of back pain, and level of serum uric acid level did not correlate with axial gout. Multiple sites may be affected in spinal gout: epidural space, intradural space, ligamentum flavum, discovertebral junction, the pedicles, facet joints, spinous processes, filum terminale, and neural foramina.

Looking back at the spine radiograph of this patient, the L2 did show erosion with overhanging edge which could represent gouty arthropathy. There are no specific MR features of gouty arthritis or tophi. The commonly described MRI features of tophi are hypointense on T1WI, hyperintense on T2WI, and heterogeneously enhanced post contrast [19, 20]. Gouty arthritis can affect any part of the vertebrae [21] with various imaging features including spondylodiscitis, destructive arthritis, or even an epidural mass [22]. The enhancing epidural components mimic an epidural mass, which was also described by Wendling *et al*. [22] and Hou *et al.* [5]. Our patient demonstrated hypointensity on T1W1 which correlates with endplate sclerosis on the radiograph (Fig. 3) and heterogenous enhancement post contrast (Fig. 4). These findings are similar to an infective cause of spondylodiscitis.

Treatment for spinal tophi depends on the clinical presentation. The presence of neurological impairment warrants surgical intervention. In many cases, the diagnosis of spinal tophi was not suspected and surgery was performed for presumed epidural abscess/spondylodiscitis/spinal mass. In some cases, a fine-needle aspiration or biopsy was performed and once the diagnosis of spinal tophi was obtained, medical treatment was started [9, 19, 23]. Medical treatments for gout include colchicine, NSAIDs, and steroids for acute gout attack, followed by urate-lowering therapies such as allopurinol, febuxostat, or probenecid.

## Conclusion

Spinal tophi are rare. The presentation may be varied and diagnosis is difficult as it may mimic other conditions such as epidural abscess, spondylodiscitis, and neoplasm. The imaging features are non-specific and when neurological signs are present, surgical intervention may be needed. Medical treatment may be instituted when neurological signs are absent.
